# Dysregulated expression of MIG/CXCL9, IP-10/CXCL10 and CXCL16 and their receptors in systemic sclerosis

**DOI:** 10.1186/ar3242

**Published:** 2011-02-08

**Authors:** Bradley J Rabquer, Pei-Suen Tsou, Yong Hou, Eshwar Thirunavukkarasu, G Kenneth Haines, Ann J Impens, Kristine Phillips, Bashar Kahaleh, James R Seibold, Alisa E Koch

**Affiliations:** 1 Department of Internal Medicine, University of Michigan Medical School, 109 Zina Pitcher Dr., Ann Arbor, MI 48109, USA; 2 Department of Pathology, Yale University, 200 South Frontage Rd., New Haven, CT 06510, USA; 3 Department of Medicine, University of Toledo, 3000 Arlington Ave., Toledo, OH 43614, USA; 4 Current address: Scleroderma Research Consultants, LLC, 97 Deer Run, Avon, CT 06001, USA; 5 Department of Veterans Affairs, VA Medical Service, 2215 Fuller Rd., Ann Arbor, MI 48105, USA

## Abstract

**Introduction:**

Systemic sclerosis (SSc) is characterized by fibrosis and microvascular abnormalities including dysregulated angiogenesis. Chemokines, in addition to their chemoattractant properties, have the ability to modulate angiogenesis. Chemokines lacking the enzyme-linked receptor (ELR) motif, such as monokine induced by interferon-γ (IFN-γ) (MIG/CXCL9) and IFN-inducible protein 10 (IP-10/CXCL10), inhibit angiogenesis by binding CXCR3. In addition, CXCL16 promotes angiogenesis by binding its unique receptor CXCR6. In this study, we determined the expression of these chemokines and receptors in SSc skin and serum.

**Methods:**

Immunohistology and enzyme-linked immunosorbent assays (ELISAs) were used to determine chemokine and chemokine receptor expression in the skin and serum, respectively, of SSc and normal patients. Endothelial cells (ECs) were isolated from SSc skin biopsies and chemokine and chemokine receptor expression was determined by quantitative PCR and immunofluorescence staining.

**Results:**

Antiangiogenic IP-10/CXCL10 and MIG/CXCL9 were elevated in SSc serum and highly expressed in SSc skin. However, CXCR3, the receptor for these chemokines, was decreased on ECs in SSc vs. normal skin. CXCL16 was elevated in SSc serum and increased in SSc patients with early disease, pulmonary arterial hypertension, and those that died during the 36 months of the study. In addition, its receptor CXCR6 was overexpressed on ECs in SSc skin. At the mRNA and protein levels, CXCR3 was decreased while CXCR6 was increased on SSc ECs vs. human microvascular endothelial cells (HMVECs).

**Conclusions:**

These results show that while the expression of MIG/CXCL9 and IP-10/CXCL10 are elevated in SSc serum, the expression of CXCR3 is downregulated on SSc dermal ECs. In contrast, CXCL16 and CXCR6 are elevated in SSc serum and on SSc dermal ECs, respectively. In all, these findings suggest angiogenic chemokine receptor expression is likely regulated in an effort to promote angiogenesis in SSc skin.

## Introduction

Systemic sclerosis (scleroderma, SSc) is a multisystem disorder that is characterized by fibrosis of the skin and internal organs, early inflammation, and vascular alterations. As the disease progresses, a loss of vasculature is observed in many organs, including the skin [1]. However, despite the loss of vasculature, compensatory angiogenesis is dysregulated and does not occur normally [2].

Angiogenesis is a highly regulated process of new blood vessel formation from pre-existing vessels. It is initiated by either proangiogenic mediators which promote the release of proteolytic enzymes or those that activate endothelial cells (ECs), inducing proliferation or migration [3]. Several types of proangiogenic mediators have been identified including growth factors, cytokines, and chemokines.

Chemokines are a family of small proteins that have leukocyte activation and chemoattractant properties. In addition, we and others have shown that some chemokines modulate angiogenesis [4,5]. CXC chemokines containing the ELR motif (Glu-Leu-Arg), such as interleukin-8 (IL-8/CXCL8), are potent angiogenic factors [5]. In addition, CXCL16 is a proangiogenic chemokine that promotes angiogenesis by binding CXCR6 on the surface of ECs [6]. By contrast, CXC chemokines lacking the ELR motif, including monokine induced by interferon-γ (MIG/CXCL9) and interferon-γ inducible protein 10 (IP-10/CXCL10), are natural inhibitors of angiogenesis [5]. These chemokines inhibit angiogenesis by binding CXCR3 on the surface of ECs [7].

In SSc, previous studies have suggested a net increase in proangiogenic factors locally in the skin and systemically in the serum, including the overexpression of select chemokines [8]. Of these, several proangiogenic chemokines are upregulated in SSc serum including IL-8/CXCL8, growth-regulated oncogene-α (Gro-α/CXCL1), and monocyte chemoattractant protein-1 (MCP-1/CCL2) [9-14]. In addition, potent antiangiogenic chemokines such as platelet factor 4 (PF4/CXCL4) [15] and IP-10/CXCL10 [16] have been shown to be upregulated in SSc serum. However, the expression of their receptors has not been examined. Therefore, we examined the expression of antiangiogenic MIG/CXCL9 and IP-10/CXCL10, and proangiogenic CXCL16 in SSc serum and skin, and their receptors in SSc skin and on ECs derived from the skin of patients with SSc. Our results suggest that while both pro- and antiangiogenic chemokines are elevated systemically in SSc, their receptors may be regulated in an effort to promote angiogenesis in SSc skin.

## Materials and methods

### Patients and controls

SSc patient and normal volunteer characteristics are given in Table 1. Punch biopsy skin samples (4 mm) were obtained from subjects with SSc and normal volunteers. Two biopsies were taken from SSc patients, one from the proximal arm, which was less clinically involved, and the other from the distal forearm, which was more clinically involved [17]. One biopsy was taken from the forearm of healthy control subjects. Peripheral blood samples were also collected. All SSc patients fulfilled the American College of Rheumatology criteria for classification of SSc and also met the criteria for diffuse SSc [18]. Biopsies were taken after informed consent, and this study was approved by the University of Michigan Institutional Review Board. Complete medical histories were also taken at the time of biopsy, which included age, disease duration, and the presence of immunomodulating therapy. Clinical symptoms were defined as: interstitial lung disease = ground glass opacification or evidence of pulmonary fibrosis by high resolution computed tomography; renal disease = history of hypertensive scleroderma renal crisis; pulmonary arterial hypertension = determined by right heart catheterization; digital ulcers = ischemic ulcer on digital tip; gastrointestinal disease = esophageal dysmotility or small intestinal dysmotility; gastric antral vascular ectasia = diagnosed by endoscopy. Patients were also stratified according to early (less than five years) or late (greater than five years) disease and those that died during the 36-month study.

**Table 1:** SSc patient and normal volunteer characteristics

	SSc patients	Normal volunteers
Age (years)	52.5 ± 1.8^a^	51.2 ± 4.4
Women	18	3
Men	2	7
Diffuse SSc	19	NA^b^
Raynaud's	19	NA
Disease duration (years)	3.7 ± 0.8	NA
Early disease^c^	13	NA
Late disease	7	NA
Deceased	4	NA
Renal involvement	1	NA
Interstitial lung disease	10	NA
Pulmonary arterial hypertension	2	NA
Digital ulcers	10	NA
Gastrointestinal disease	19	NA
Gastric antral vascular ectasia	3	NA
Immunomodulatory therapy	3	NA
SSc patients serum CXCL16 (ng/ml)		
	With	Without
Pulmonary arterial hypertension	5.7 ± 1.2 (*n *= 2)^d^	4.5 ± 0.1 (*n *= 18)
Early disease	4.9 ± 0.2 (*n *= 13)^d^	4.0 ± 0.1 (*n *= 7)
Deceased	5.6 ± 0.4 (*n *= 4)^d^	4.4 ± 0.1 (*n *= 16)

^a^Mean ± SEM. ^b^NA = Not applicable. ^c^Early disease = less than five years. ^d^*P *< 0.05.

### Immunohistology

We performed immunohistologic staining on cryosections from SSc and normal skin, as described previously [19]. Antibodies against MIG/CXCL9 (R&D Systems, Minneapolis, MN, USA), IP-10/CXCL10 (Peprotech, Rocky Hill, NJ, USA), CXCL16 (Peprotech), CXCR3 (R&D Systems), and CXCR6 (R&D Systems) were used as primary antibodies. Purified nonspecific isotype matched IgG was used as a control. An antibody against von Willebrand factor (vWF) was used to confirm the presence of ECs in normal and SSc skin sections as previously described [17]. Staining was evaluated in duplicate under blinded conditions and graded by a pathologist. Entire tissue sections were examined for cellular immunoreactivity and cell types were distinguished based on their characteristic morphology. For quantification, the percentage of positive cells was calculated as stained cells in proportion to all cells of a distinctive subset.

### Enzyme-linked immunosorbent assays (ELISAs)

Commercial ELISA kits were purchased and used following the manufacturer's instructions to determine the concentrations of IP-10/CXCL10 (Invitrogen, Carlsbad, CA, USA), CXCL16 (Peprotech), and MIG/CXCL9 (R&D Systems) in SSc and normal serum.

### Isolation of ECs from SSc skin

Microvascular ECs from SSc skin were isolated as previously described [20,21]. Briefly, the epidermis and subcutaneous layers were mechanically removed from skin biopsies, leaving the vascularized dermal layer. Microvascular colonies were selected and ECs were positively selected using Dynabeads CD31 (Invitrogen). Confirmation of EC selection was made using antibodies against CD31 and von Willebrand factor (vWF).

### SSc EC and human dermal microvascular endothelial cell (HMVEC) cell culture

HMVECs were obtained from Lonza (Basel, Switzerland) and cultured using complete EC basal medium (EBM)-2 medium with 5% FBS and EC growth medium-2 SingleQuots (Lonza) as previously described [22]. SSc ECs were cultured in complete EBM-2 media with EC growth medium-2 SingleQuots with 20% FBS. HMVECs and SSc ECs were used between passage 5 and 11. SSc ECs and HMVECs were plated on gelatin coated six-well plates and grown to confluence. The cells were serum starved overnight in EBM media with 0.1% FBS prior to RNA isolation.

### RNA isolation and quantitative PCR (qPCR)

Total RNA was extracted from SSc ECs and HMVECs and qPCR was performed using a Mastercycler ep realplex (Eppendof, Hauppauge, NY, USA) as previously described [23]. The primer sets used were CXCR3 5'TGGCCGAGAAAGCAGGG3' and 5'AGGCGCAAGAGCAGCATC3'; CXCR6 5'ATGGCAATGTCTTTAATCTCGACAA3' and 5'TGAAAGCTGGTCATGGCATAGTATT3'; and CXCL16 5'ACTACACGAGGTTCCAGCTCC3' and 5'CTTTGTCCGAGGACAGTGATC3' [24,25]. Primers specific for β-actin were used as a control.

### Immunofluorescence

Immunofluorescence staining was performed as previously described [26]. Primary antibodies specific for vWF (Dako, Glostrup, Denmark), CXCR3 (R&D Systems), CXCR6 (R&D Systems), or mouse IgG (ThermoFisher, Waltham, MA, USA) (10 μg/ml) were used. Binding was detected using Alexa Fluor 555-conjugated donkey anti-mouse antibody (Molecular Probes, Eugene, OR, USA) and 4',6-diaminido-2-phenylindole (DAPI, Molecular Probes) nuclear stain was added to observe nuclei. Staining was detected using an Olympus fluorescence microscope (Olympus America, Melville, NY, USA). Images were taken at 400×.

### Statistical analysis

Student's *t*-tests and, where appropriate, ANOVAs were performed, and *P*-values less than 0.05 were considered significant. All values presented were the mean ± standard error of the mean (SEM).

## Results

### Chemokine expression in SSc serum

**Figure 1:**
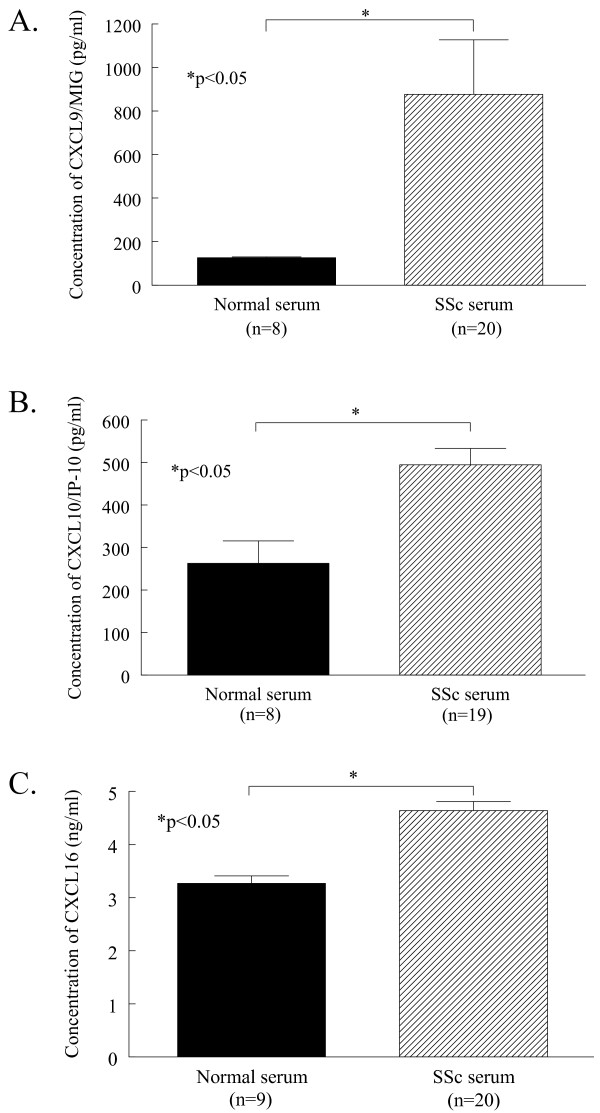
**MIG/CXCL9, IP-10/CXCL10, and CXCL16 are overexpressed in SSc serum**. The amounts of MIG/CXCL9 (**A**), IP-10/CXCL10 (**B**), and CXCL16 (**C**) were determined in normal and SSc serum by ELISA. Means are given with SEM. Differences were determined using the Student's *t*-test and *P-*values less than 0.05 were significant. *n *= number of patients.

Serum from patients with SSc and healthy volunteers was assayed using ELISAs to determine the expression of pro- and antiangiogenic chemokines. SSc serum had significantly elevated levels of antiangiogenic chemokines compared to normal controls (Figure 1A). The expression of MIG/CXCL9 was significantly greater in SSc serum (mean 876 pg/ml ± SEM 250 pg/ml) compared to normal serum (126 pg/ml ± 1, *P *< 0.05). In addition, IP-10/CXCL10 was significantly elevated in SSc serum (495 pg/ml ± 38) compared to normal serum (263 pg/ml ± 53, *P *< 0.05, Figure 1B). Similar results were found when a representative proangiogenic chemokine was assayed in SSc and normal serum. The expression of CXCL16 was significantly greater in SSc serum (4.6 ng/ml ± 0.2) compared to normal serum (3.3 ng/ml ± 0.1, *P *< 0.05, Figure 1C). These results demonstrate that both pro- and antiangiogenic chemokines are significantly upregulated in the serum of patients with SSc.

In patients with SSc, CXCL16 was significantly elevated in those with pulmonary arterial hypertension (5.7 ng/ml ± 1.2, *n *= 2) compared to those without (4.5 ng/ml ± 0.1, *n *= 18, *P *< 0.05, Table 1). CXCL16 was also significantly increased in SSc patients that died during the 36 months of the study (5.6 ng/ml ± 0.4, *n *= 4) compared to surviving patients (4.4 ng/ml ± 0.1, *n *= 16, *P *< 0.05). Moreover, SSc patients with early disease had significantly increased levels of CXCL16 (4.9 ng/ml ± 0.2, *n *= 13) compared to those with late disease (4.0 ng/ml ± 0.1, *n *= 7, *P *< 0.05). However, no differences were observed in any clinical characteristics with respect to serum MIG/CXCL9 and IP-10/CXCL10.

### Chemokine and chemokine receptor expression in SSc skin

**Figure 2:**
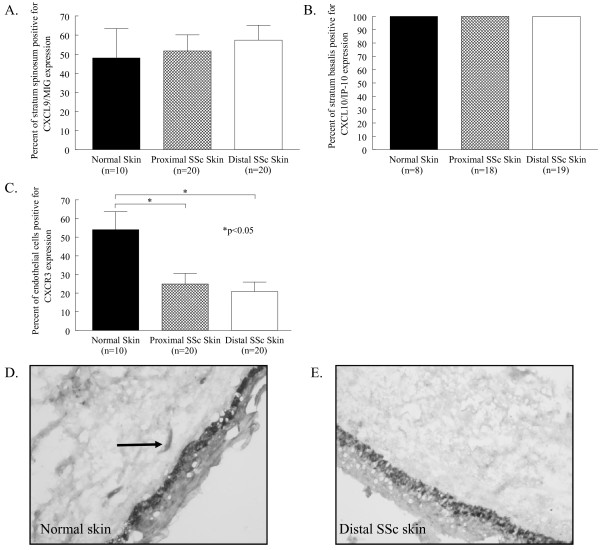
**MIG/CXCL9 and IP-10/CXCL10 are highly expressed, but CXCR3 is decreased, in SSc and normal skin**. Frozen sections of proximal and distal SSc and normal skin were stained for MIG/CXCL9, IP-10/CXCL10, or CXCR3. (**A**), MIG/CXCL9 was highly expressed in the stratum spinosum of normal (48%), proximal SSc (52%), and distal SSc (57%) skin. (**B**), IP-10/CXCL10 was uniformly expressed in the stratum basalis of normal, proximal SSc, and distal SSc skin at 100%. (**C**), CXCR3 was significantly decreased on ECs in proximal SSc (25%) and distal SSc skin (21%) compared to normal skin (54%). Representative photos of CXCR3 immunohistological staining in normal (**D**) and distal SSc skin (**E**) are shown. Arrows indicate positive staining. Means are given with the SEM. *n *= the number of patients. *P *< 0.05 was considered significant.

After finding elevated expression of the antiangiogenic chemokines MIG/CXCL9 and IP-10/CXCL10 in SSc serum, we sought to determine their expression, and the expression of their receptor CXCR3, in SSc skin (Figure 2). MIG/CXCL9 was primarily expressed in the stratum spinosum of the epidermis in normal skin (48% ± 15) and both proximal (52% ± 9) and distal SSc skin (57% ± 8), with no significant differences observed among the three groups. MIG/CXCL9 was also expressed at much lower quantities on normal and SSc dermal ECs and fibroblasts (data not shown). Similarly, IP-10/CXCL10 was also highly expressed in the epidermal layer stratum basalis in normal skin and proximal and distal SSc skin (all 100%, Figure 2B). In contrast to MIG/CXCL9, the expression of IP-10/CXCL10 was not observed on any other cell types in either normal or SSc skin. Importantly, while MIG/CXCL9 and IP-10/CXCL10 were elevated systemically and highly expressed locally in SSc skin, the expression of their receptor CXCR3 was decreased (Figure 2C). CXCR3 expression was restricted to ECs, and was significantly decreased on ECs in both proximal (25% ± 6) and distal SSc skin (21% ± 5) compared to normal skin (54% ± 10, *P *< 0.05). These findings suggest that ECs in SSc may have a diminished response to these chemokines based on the downregulation of their receptor.

**Figure 3:**
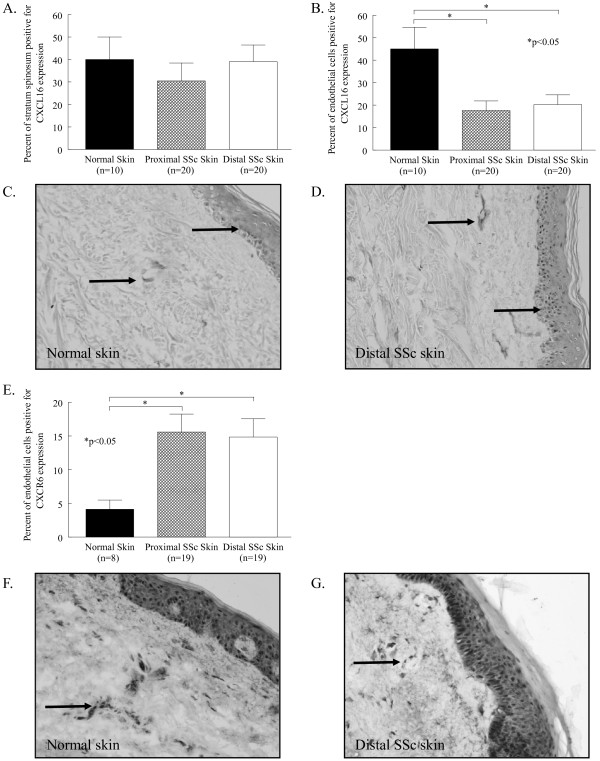
**CXCL16 and CXCR6 are highly expressed in SSc compared to normal skin**. Frozen sections of proximal and distal SSc and normal skin were stained for CXCL16 or CXCR6. (**A**), CXCL16 was highly expressed in the stratum spinosum of normal (40%), proximal SSc (30%), and distal SSc (39%) skin. (**B**), CXCL16 was significantly decreased on ECs in proximal SSc (18%) and distal SSc skin (20%) compared to normal skin (45%). Representative photos of CXCL16 immunohistological staining in normal (**C**) and distal SSc skin (**D**) are shown. (**E**), CXCR6 was significantly increased on ECs in proximal SSc (16%) and distal SSc skin (15%) compared to normal skin (4%). Representative photos of CXCR6 immunohistological staining in normal (**F**) and distal SSc skin (**G**) are shown. Arrows indicate positive staining. Means are given with the SEM. *n *= the number of patients. *P *< 0.05 was considered significant.

We also examined the expression of the proangiogenic chemokine CXCL16 in normal and SSc skin (Figure 3). CXCL16 was highly expressed in the stratum spinosum of the epidermis of normal skin (40% ± 10) and proximal (30% ± 8) and distal SSc skin (39% ± 8). CXCL16 was also expressed on dermal ECs, with its expression significantly decreased on proximal (18% ± 4) and distal SSc skin (20% ± 4) compared to normal skin (45% ± 10, *P *< 0.05, Figure 3B). Moreover, the expression of its exclusive receptor, CXCR6, was significantly elevated on ECs in both proximal (16% ± 3) and distal SSc skin (15% ± 3) compared to normal skin (4% ± 1, *P *< 0.05, Figure 3C). In contrast with the expression of the antiangiogenic receptor CXCR3, proangiogenic CXCR6 was upregulated on ECs in SSc skin.

### Chemokine and chemokine receptor expression by SSc ECs

After determining the expression of pro- and antiangiogenic chemokines in SSc skin, we isolated ECs from SSc skin biopsies and examined chemokine receptor expression at the mRNA level [20]. SSc ECs and HMVECs were cultured, their mRNA extracted, cDNA prepared, and quantitative PCR was performed. The mRNA expression of CXCR3 was decreased and CXCR6 was increased in SSc ECs, similar to our immunohistological findings using whole skin biopsies (Table 2).

**Table 2:** CXCR3 is decreased while CXCR6 is increased on SSc ECs

	**Average relative abundance**^**a**^		
		
	**HMVEC**^**b**^	**SSc EC**^**c**^	Ratio
CXCR3	3.8 E-04 ± 9.1 E-06	1.5 E-04 ± 8.8 E-05	0.4
CXCR6	2.2 E-05 ± 1.1 E-05	3.2 E-05 ± 8.1 E-06	1.5
CXCL16	1.1 E-05 ± 1.6 E-06	1.4 E-04 ± 3.7 E-05	13.4

^a^Chemokine or chemokine receptor/β-actin. ^b^*n *= three replicates. ^c^*n *= three patient derived lines. ^d^Mean ± SEM

**Figure 4:**
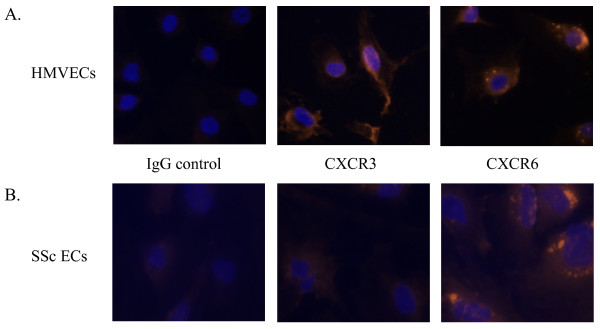
**CXCR3 expression is decreased while CXCR6 expression is unchanged on SSc ECs compared to HMVECs**. HMVECs (**A**) and SSc ECs (**B**) were cultured on gelatin-coated chambers and immunofluorescence stained. Staining with specific antibodies against CXCR3, CXCR6, or irrelevant IgG control is shown. Nuclei were counter stained with DAPI. Staining was detected using a fluorescence microscope and images were taken at 400×.

After finding that EC CXCR3 and CXCR6 were downregulated and upregulated, respectively, by immunohistochemistry and quantitative PCR, we performed immunofluorescence staining on cultured SSc ECs to visualize the protein expression of these antigens *in vitro*. We found that cultured SSc ECs had reduced expression of the endothelial maker vWF as previously described (data not shown) [27]. In addition, SSc ECs showed reduced staining for CXCR3, while the expression of CXCR6 was not notably increased in SSc ECs (Figure 4).

## Discussion

Angiogenesis in SSc skin does not occur normally despite the overexpression of proangiogenic mediators in the skin and serum [2]. Select chemokines, in addition to their role as chemoattractant molecules, modulate angiogenesis. In general, CXC chemokines containing an ELR motif are angiogenic, while CXC chemokines lacking the motif are angiostatic [5]. Several other chemokines have been shown to be proangiogenic including MCP-1/CCL2, macrophage inflammatory protein-1α (MIP-1α/CCL3), and CXCL16 [7]. Collectively, these and other studies established the role of chemokines in angiogenesis.

Angiogenic chemokines have previously been associated with SSc pathogenesis [8]. We and others have shown that IL-8/CXCL8 is a potent proangiogenic chemokine overexpressed in both SSc serum and skin [9,11,14,28]. In addition, it has been associated with SSc lung pathology, as it is elevated in pulmonary bronchial lavage fluid and secreted by alveolar macrophages [29]. Moreover, the angiogenic CXC chemokines Gro-α/CXCL1 and stromal derived factor-1 (SDF-1/CXCL12) have also been found to be upregulated in SSc serum [9,10,30]. Among CC chemokines that are angiogenic, MCP-1/CCL2 and MIP-1α/CCL3 have been shown to be upregulated in SSc [10,12,29]. PF4/CXCL4, which by contrast is an antiangiogenic chemokine, has also been shown to be upregulated in SSc serum [15].

In this study, we examined the expression of select pro- and antiangiogenic chemokines and their receptors in SSc. We found that antiangiogenic MIG/CXCL9 was significantly elevated in SSc serum compared to normal controls. The only other study to examine its expression in SSc serum found that MIG/CXCL9 was detected at similar rates in diffuse and limited SSc serum and normal control serum [16]. However, the assays employed in that study were not as sensitive as the methods used here, as their analysis was limited to detection, whereas we were able to quantify MIG/CXCL9 expression. We also found that IP-10/CXCL10 was elevated in SSc serum compared to normal controls. Similar to our results, Fujii *et al. *demonstrated that IP-10/CXCL10 was detected more often in SSc serum compared to healthy control serum [16]. In addition, a more recent study has demonstrated that IP-10/CXCL10 is significantly elevated in early SSc serum compared to controls, and that its level significantly decreases after five years [31]. Moreover, the level of IP-10/CXCL10 correlated with lung and kidney involvement in SSc patients [31].

In accordance with these findings, we found that both MIG/CXCL9 and IP-10/CXCL10 were highly expressed in the epidermis. Mediators produced by keratinocytes in the epidermis may cross into the dermal layer and affect the function of fibroblasts and other cell types in the dermis [32]. This suggests that chemokines expressed in the epidermis may act on dermal vasculature, along with the circulating chemokines in the serum.

After examining the expression of the antiangiogenic chemokines MIG/CXCL9 and IP-10/CXCL10 in SSc skin and serum, we then sought to determine the expression of their receptor. The ELR negative chemokines PF4/CXCL4, MIG/CXCL9, and IP-10/CXCL10 mediate their leukocyte chemoattractant and angiogenic effects by interacting with CXCR3 [7]. To date, three splice variants of CXCR3 have been identified; CXCR3-A, CXCR3-B, and CXCR3-alt [33]. However, the angiostatic effects of PF4/CXCL4, MIG/CXCL9, and IP-10/CXCL10 are mediated solely by CXCR3-B [34,35]. We found that CXCR3 is decreased on ECs in both proximal and distal SSc skin compared to normal skin. Moreover, we observed that SSc ECs have reduced mRNA levels of CXCR3 compared to HMVECs, and that *in vitro *CXCR3 cell surface expression is decreased on SSc ECs compared to HMVECs. Previous studies describing the expression of CXCR3 on ECs *in vitro *have yielded inconsistent results. CXCR3 has been shown to be expressed on HMVECs, but not on human umbilical vein ECs (HUVECs) [33,36]. This may be due to the different nature of microvascular ECs and venous ECs, as Chi *et al. *has previously shown that ECs from different anatomical sites have disparate gene expression profiles [37]. Thus HMVECs isolated from adult skin are a more suitable control population for our SSc ECs than HUVECs. In addition, differences in cytokine and growth factor expression may alter CXCR3 expression, as tumor necrosis factor (TNF)-α downregulates CXCR3 expression [38]. TNF-α and other proinflammatory cytokines are known to be upregulated in SSc skin and serum, which may account for the decreased CXCR3 expression we observed *in vitro *and *in vivo *[12,39,40].

In addition, we also determined the expression of the proangiogenic receptor CXCR6 and its ligand CXCL16. CXCL16 is a recently described CXC chemokine that is found in both membrane bound and soluble forms, the latter following cleavage by a disintegrin and metalloproteinase 10 (ADAM10) [41]. It has a unique receptor in CXCR6, which the membrane bound form of CXCL16 binds to facilitate firm adhesion of emigrating leukocytes, and the soluble form utilizes to mediate its chemotactic properties [42]. CXCL16 is expressed on a number of different cell types, including leukocytes, ECs and keratinocytes [43].

We found that CXCL16 was significantly upregulated in SSc serum. In addition, it was the only chemokine in our study that was associated with specific SSc clinical features. Serum CXCL16 was elevated in SSc patients that died between the time of biopsy and the time of data analysis, and in those with pulmonary arterial hypertension and early disease. A recent study by Yanaba *et al. *found similar results and showed that CXCL16 serum levels correlated with the extent of skin sclerosis [43]. In addition, they demonstrated that CXCL16 levels decrease as SSc skin sclerosis improves [43]. Collectively, these findings implicate CXCL16 as a chemokine of interest in SSc that warrants further study.

In SSc skin biopsies, we found that CXCL16 was highly expressed in the stratum spinosum layer of the epidermis, and decreased on dermal ECs compared to normal skin. In contrast, our *in vitro *results indicate that CXCL16 mRNA is modestly increased in SSc ECs. It is likely that this discrepancy is caused by the differences in detection techniques, and the fact that CXCL16 exists in both secreted and membrane bound forms.

The receptor for CXCL16, CXCR6, is expressed by several cell types, most notably memory and activated T cells, cancer cells, and ECs [44]. We observed that CXCR6 was significantly upregulated on dermal ECs in both proximal and distal SSc skin biopsies. Moreover, we found a similar expression pattern on cultured SSc ECs compared to HMVECs. To date, little is known regarding the regulation of CXCR6 expression. However, hypoxia has been shown to be a strong inducer of CXCR6 expression via hypoxia-inducible factor-1α [6]. Hypoxia is a characteristic feature of SSc [45]. In SSc, the hypoxic environment is thought to be a factor in the overexpression of potent angiogenic factors such as VEGF [45]. Likewise, it seems probable that skin hypoxia could result in an increase of CXCR6 expression on SSc ECs. In addition, CXCR6-expressing HUVECs migrate in response to CXCL16 [6]. As EC chemotaxis is an initial step in the angiogenic cascade, these findings suggest that CXCL16/CXCR6 may be important in mediating SSc angiogenesis.

## Conclusions

Diminished dermal vasculature and dysregulated angiogenesis are hallmarks of SSc. Our results indicate a systemic overexpression of proangiogenic CXCL16 and antiangiogenic MIG/CXCL9 and IP-10/CXCL10 in SSc serum. However, differential expression of CXCR3 and CXCR6 was observed on SSc dermal ECs. Collectively, these results argue that despite high serum levels of antiangiogenic chemokines, the downregulation of their receptor on SSc ECs prevents them from directly contributing to the hypovascular state of SSc skin. In addition, the increase of CXCR6 on SSc ECs points to CXCL16 being a relevant angiogenic factor in SSc that warrants further study.

## Abbreviations

ADAM10: a disintegrin and metalloproteinase 10; DAPI: 4',6-diaminido-2-phenylindole; ECs: endothelial cells; ELISA: enzyme-linked immunosorbent assay; Glu-Leu-Arg: ELR motif; Gro-α/CXCL1: growth-regulated oncogene-α; HMVECs: human microvascular endothelial cells; HUVECs: human umbilical vein ECs; IFN-γ: interferon-γ; IL: interleukin; IP-10/CSCL10: IFN-inducible protein 10; MCP-1/CCL2: monocyte chemoattractant protein-1; MIG/CXCL9: monokine induced by IFN-γ; MIP-1α/CCL3: macrophage inflammatory protein-1α; PF4/CXCL4: platelet factor 4; qPCR: quantitative polymerase chain reaction; SDF-1/CXCL12: stromal derived factor-1; SEM: standard error of the mean; SSC: systemic sclerosis; vWF: von Willebrand factor.

## Competing interests

The authors declare that they have no competing interests.

## Authors' contributions

BJR conceived the study, and participated in the immunohistology, ELISA, cell culture, qPCR, immunofluorescence, and data analysis and drafted the manuscript. PT participated in the immunohistology, ELISA, qPCR, and data analysis. YH participated in the immunohistology and ELISAs. ET participated in the immunohistology and ELISAs. GKH scored the immunohistology. AJI performed the statistical analysis. KP conceived the study and collected clinical data. BK isolated ECs from SSc skin. JRS conceived the study and collected clinical data. AEK conceived the study and drafted the manuscript. All authors read and approved the final manuscript.
